# The communication aspects of the Ebola virus disease outbreak in Western Africa – do we need to counter one, two, or many epidemics?

**DOI:** 10.3325/cmj.2015.56.496

**Published:** 2015-10

**Authors:** Joachim Allgaier, Anna Lydia Svalastog

**Affiliations:** 1 Institute of Science, Technology and Society Studies, Alpen-Adria-University, Klagenfurt, Austria; 2 Psychosocial Work, Department for Health and Social Studies, Østfold University College, Halden, Norway

In public health crises such as the outbreak of the Ebola virus disease in West Africa in 2014, the key role in saving the lives of infected people is played by the management and dissemination of scientific and medical knowledge. After the World Health Organization (1) has announced that no confirmed cases of the Ebola virus disease were reported in the week to October 4, 2015, we wanted to analyze various communications and communication strategies concerning the Ebola epidemic in West Africa. Our argument is that for effective health communication strategies in public health crises it is crucial to respond to local contexts and to take them into account on various levels. We are going to lay out our argument using illustrative examples.

## Information spread during disease outbreak

During the early stages of disease outbreaks we generally find a great deal of uncertainty about the facts. The new public health situation still needs to be assessed by professionals, while the infection accompanied by all kinds of news and rumors travels fast. Confirmation of facts is not always immediately possible and scientific information and peer reviewed-literature is always lagging behind the news reports and rumors. This is what makes social online networks, mobile phones communication, and radio crucial sources of information in the early stages of epidemics (2). The credibility and assessment of information is also affected by social and personal relations between people. This is a reason why we are focusing on coverage about Ebola in news and social online media. Of course, there is also a lot of room for speculation about causes of and potential cures for disease outbreaks when a story about an infectious epidemic breaks.

Because African countries are not always in the spotlight of Western media it took some time until the Ebola outbreak became a global news story. When there are no authoritative sources (such as journalistic or scientific accounts) around, rumors can take hold and miraculous remedies are proposed. In addition, present and past events and politics frame the understandings of the Ebola and other disease outbreaks (3). What at a distance and in retrospect is defined as misunderstandings and wrongdoings, may in a particular context, appear as reasonable. However, there is hope that more published news stories will improve the available information, consensus, and knowledge base. We think that it is also important to take the present and past cultural contexts into account not only to counter misunderstandings, but also to avoid reinforcement of (post-)colonial images of the ignorant or uncivilized natives of the African continent.

## More than a virus epidemic

Our argument is that when we want to deal effectively with physical local disease outbreaks and the situation on the ground, there are various forms of epidemics that need to be tackled. For instance, Victor Luckerson (4) describes the complications caused by fear and misinformation spread in online social media networks such as Facebook or Twitter in the fight against Ebola. Far away from West Africa, in Iowa the Department of Public Health was forced to issue a statement dispelling rumors that Ebola had arrived in Iowa and spread among its citizens. Lots of posts in social media networks also claimed that Ebola can spread through the air, water, or food, which does not correspond with the scientific information on the subject. In this news article (4), the spread of “incorrect” information about Ebola was compared to an online virus, where “infected” internet users keep on “infecting” others with “incorrect” information, which is then further disseminated via tweets or Facebook posts. People tend to believe the information from people they know, which makes it particularly difficult for health authorities to counter misinformation. Social media clearly helped to spread rumors and unscientific information about diseases not just locally in the concerned African countries, but also in other parts of the world (5,6).

These conspiracy theories sometimes also resulted in local mistrust and even hostility toward help from foreign health workers and NGOs. In addition, they do not only stigmatize Ebola survivors but also Ebola help workers and medical professionals. The outbreak of a biological virus therefore corresponds to the outbreak of informational viruses that entail rumors. These are contagious as well, spreading through tabloid newspapers, lurid TV reports, and a plethora of online channels, mutating, multiplying and adapting to new contexts. The spread of the informational viruses often interferes with the fight against actual biological viruses.

While in the United States public health authorities have means, experience, and strategies to counter health-related rumors, this is much more difficult in the African regions affected by Ebola. The local state authorities are often negatively perceived among the population and many locals do not consider them as being trustworthy sources. Some conspiracy theories actually accuse local governments to have set things in motions to get access to aid money or gain votes in the elections. Local health and science communicators need to be more inventive to reach out to the local public and cannot rely on state authorities alone. There are some noteworthy examples where various individuals took matters in their own hands to inform their fellow citizens via YouTube. For instance, some Liberians posted videos on proper hand washing on YouTube, and a Liberian rapper named Shadow made music videos called “Ebola in Town,” which cautions against touching and kissing and links Ebola to eating bush meat. The video quickly reached more than 100 000 views (7).

Social media are not only important for the locals in West Africa, but they also connect expats with friends and relatives back home. They therefore also function as feedback channels for receiving information from abroad from people one knows personally. West Africans in the United States, for instance, also used Facebook as a fundraising tool. Liberian advocacy groups posted audio announcements about Ebola in local native languages on Facebook to reach those who do not understand English and those who cannot read. The fundraising also made it possible to run the announcements on a local Liberian radio station (7). International organizations like the Centre for Disease Control (CDC) in the United States and the World Health Organization (WHO) found it difficult to respond to rumors about miracle curses against Ebola advertised in various news outlets online, because they lacked the trust and credibility in the eyes of many locals. It has been particularly difficult for them to make their voice heard locally. However, in due course they learned that they have to rely on locals to spread and amplify their messages (7,8).

Especially in the beginning of an outbreak, media and internet coverage is very unreliable and potentially harmful. For instance, Information Week (8) reported that various potential remedies, cures, and precautions against Ebola infection – including eating raw onion, kola nut, or chocolate, or drinking coffee – were advertised online. In Nigeria at least two people died and more were hospitalized because they had applied harmful solutions for countering Ebola. From a medical point of view having people hospitalized because of harmful and unscientific rumors does clearly demand action. Consequently, Nigerian information minister Labaran Maku had to issue a statement in mid-August 2014 that drinking lots of salt water would not cure Ebola (9). In Liberia the information minister Lewis Brown issued updates during a regular “Ebola Hour,” also posted on YouTube, to provide the public and health workers in Liberia with accurate facts (9).

## The importance of the context

It was also reported that the people in the affected regions have become distrustful of doctors from the West. The rumors and misinformation in a combination with fear of contagion had apparently led to attacks on some health workers and blocking their access to treat infections. Some conspiracy theories claimed that Ebola was brought to the region on purpose by Westerners and that now they were looking for infected people in order to kill them (5,6). The aid workers suddenly had two enemies to face: the Ebola epidemic and the fear that had produced hostility. This hostility has made it even more difficult to deal with Ebola treatments and care. For instance, members of Médecins Sans Frontiėres found that locals were hiding their sick from them and prevented humanitarian organizations to do their work (10). Various news stories also surfaced that health and aid workers were physically attacked. For instance, a particularly shocking story reported that in the village of Womey in South-East Guinea a small team of health workers and journalists were stoned to death by angry residents (10).

These severe problems led international organizations working in Guinea to bring in local anthropologists to analyze the situation and improve the cooperation with local communities. The anthropologists found that the treatment of Ebola had so far strongly focused on the biomedical aspects alone and disregarded parameters such as community, society, and culture. Consequently, they started to take the fears and concerns of the members of local communities seriously, also taking traditional beliefs and views into account. They found that terms like “isolation centers” for the locals meant “death chambers,” from where no one was seen coming out alive. As a simple first measure, the anthropologists suggested to change the term to “treatment centers” (10).

Burial customs and rituals also played an important part in spreading infections, since they involved touching the infected deceased bodies. When death and threatening situations occur, social relations and customs become even more important than usual. The acute uncertainty of everyday life highlights the importance of belonging and a sense of community. The involved anthropologists took local beliefs and customs seriously, and engaged with the elders and other respected members of local communities in order to develop new burial rituals and customs. Understanding the traditional beliefs and local concerns in the local communities enabled the anthropologists to develop solutions how to regain the trust and credibility, and to ensure cooperation with the locals.

The overall lesson learned is that following standard biomedical protocols alone is often not enough to succeed; local knowledge, beliefs, and communities must be taken into account and effective treatment plans must be adapted to local needs and environments. The biomedical concept of contagion, for instance, can strongly differ from the complex cultural conceptions of contagion in various Non-Western cultures. If these interpretations are not taken into account, infectious disease control programs within local contexts based on meaningful community participation will not be possible (11). Health workers not only have to deal with diseased individuals, but they also need to build trustful relationships with local communities. It is important to show that humanitarian actions are not intended to undermine but to secure and sustain the local communities.

## The virtue of health communication

It is of great importance to raise awareness of effective science and health communication. It is not enough to communicate hard scientific facts alone, but to know how the disseminated information is perceived: which channels can be used effectively in what contexts; who the different audiences are; and also how communicators can be trapped in historic and present power relations. For instance, the media coverage in the United States and in Europe often focused only on the situation at home and rarely on the affected people in Western Africa. The voices of local health workers on the ground were sometimes heard (12) but the Western media were often more interested in extreme stories or stories that related to the situation in Europe and the United States The coverage sometimes had neocolonial if not even racist undertones, portraying the affected African locals as backward or irrational individuals. From an African point of view, it is not at all irrational to be skeptical about help coming from the so called developed world. Many actual bad experiences have left their mark on African people. It is also advisable to rely on the support of local voices. It is crucial to get in touch with local citizens and professionals who can help establish trustful relationships with local communities.

Science and health communication strategies should be tailored to the local circumstances. For instance, the internet in Africa is accessed mainly via mobile phones and experts expect a 20-fold increase in this type of communication in the next five years. With the costs going down and the quality going up, it is expected that there will be a massive increase in online video usage (13). The online video format combines various advantages for science and health communication: it works on a visual but also on an auditory level. This means that various spoken native languages, as well as a diverse set of subtitles, could be used. The audio information can also be understood by people who have difficulty reading. However, it must be acknowledged that online video-sharing sites such as YouTube also serve as a main channel for spreading misinformation and conspiracy theories (14). Nonetheless, we would like to encourage science and health communicators to consider the high potential of the online video format also for science and health communication purposes, especially in the context of local public health crises also to counter rumors, misconceptions, and conspiracy theories.

## Treating all epidemics

It is clear that the outbreak of local virus epidemics must be fought with the best biomedical and scientific tools and knowledge available. But we also have to deal with secondary, virtual epidemics that are taking place globally in news and social online media, where various correct and incorrect information, misconceptions, and rumors can be distributed without editorial control.

Virtual viruses are a relatively recent phenomenon that differs from traditional and non-virtual rumors. So far no consensus has been reached on how to counter them most effectively. However, we want to emphasize the importance of contextual knowledge, traditional professional and critical journalism, and the benefits of alliances between different agents that are actively involved in a breakout, so that local resources and competences are both acknowledged and supported.
